# High Moisture Extrusion of Soy Protein: Investigations on the Formation of Anisotropic Product Structure

**DOI:** 10.3390/foods10010102

**Published:** 2021-01-06

**Authors:** Patrick Wittek, Nicole Zeiler, Heike P. Karbstein, M. Azad Emin

**Affiliations:** Institute of Process Engineering in Life Sciences, Chair of Food Process Engineering, Karlsruhe Institute of Technology, 76131 Karlsruhe, Germany; patrick.wittek@kit.edu (P.W.); nicole.zeiler94@gmx.de (N.Z.); heike.karbstein@kit.edu (H.P.K.)

**Keywords:** high moisture extrusion, meat analogue, plant protein, soy protein, anisotropic structure, protein–protein interactions, multiphase system

## Abstract

The high moisture extrusion of plant proteins is well suited for the production of protein-rich products that imitate meat in their structure and texture. The desired anisotropic product structure of these meat analogues is achieved by extrusion at high moisture content (>40%) and elevated temperatures (>100 °C); a cooling die prevents expansion of the matrix and facilitates the formation of the anisotropic structure. Although there are many studies focusing on this process, the mechanisms behind the structure formation still remain largely unknown. Ongoing discussions are based on two very different hypotheses: structure formation due to alignment and stabilization of proteins at the molecular level vs. structure formation due to morphology development in multiphase systems. The aim of this paper is, therefore, to investigate the mechanism responsible for the formation of anisotropic structures during the high moisture extrusion of plant proteins. A model protein, soy protein isolate, is extruded at high moisture content and the changes in protein–protein interactions and microstructure are investigated. Anisotropic structures are achieved under the given conditions and are influenced by the material temperature (between 124 and 135 °C). Extrusion processing has a negligible effect on protein–protein interactions, suggesting that an alignment of protein molecules is not required for the structure formation. Instead, the extrudates show a distinct multiphase system. This system consists of a water-rich, dispersed phase surrounded by a water-poor, i.e., protein-rich, continuous phase. These findings could be helpful in the future process and product design of novel plant-based meat analogues.

## 1. Introduction

Plant-based meat analogues have seen a steady increase in demand in recent years due to a change in consumer behavior caused by health [1,2,3], ethical [4,5,6] and sustainability aspects [7,8,9] related to meat consumption.

However, consumers do not want to compromise on the typical fibrous meat structure and texture [10,11,12,13]. To meet these needs, meat analogues produced from plant proteins try to imitate the characteristic anisotropic structure of meat [12,14] and ensure comparable protein intake.

The extrusion process has been used since the 1960s to produce these plant-based meat-like products [15,16,17]. In this process, protein-rich raw materials are mixed with water, conveyed through the extruder barrel by rotating screws while being heated and sheared, and finally pushed through a die. First research activities on meat analogues in the 1970s were concerned with low moisture extrusion of plant proteins (LME, water content < 40%) to achieve the desired anisotropic structures [18,19,20,21]. Those were followed by the first industrial approaches and research publications on high moisture extrusion (HME, water content > 40%) later in the 1970s and 1980s [22,23,24,25].

The extrudates from LME have an expanded, porous structure caused by the sudden evaporation of water at the die exit due to the high material temperatures (>100 °C) in the process. In HME, that water evaporation is prevented by cooling the protein matrix below 100 °C with a long cooling die.

Many of the publications on extruded meat analogues that followed to date use an empirical approach to relate the parameters of the extrusion process to the product properties. However, the results are specific to the process and material systems used. The transfer to other process conditions, be it material- or machine-wise, remains very difficult. For example, extrusion setups with a raw material of the same type but from different manufacturers can lead to significantly different product structures and textures, as reported for soy protein isolate [26,27] or pea protein isolate [28].

Therefore, the formation of anisotropic structures in protein extrusion needs to be better understood at a mechanistic level. Different approaches have been used to explain the structure formation and an overview is given below.

First insights into anisotropic structure formation were gained in LME of soy proteins [29,30,31]. It was suggested that the protein molecules unfold and align in the direction of flow, which results in the anisotropic product structure. The stabilization of the aligned proteins is achieved through newly developed covalent bonds, either disulfide [30,31,32,33,34,35] or isopeptide bonds [29,36]. Later studies on HME adapted the theory of protein alignment and reported correlations between the changes in protein–protein interactions and the product structure [27,37,38,39,40,41,42].

In the 1990s, Tolstoguzov [43,44] suggested another mechanism responsible for the formation of anisotropic structures: the existence of a multiphase system. The model is derived from the research of Tolstoguzov and coworkers [45,46,47] on spinneretless spinning. It has been observed that mixtures of biopolymers in solution, protein–protein or protein–polysaccharide, develop phase separation above a certain concentration threshold, caused by thermodynamic immiscibility of the biopolymers involved; it was assumed that this phase separation would also occur at higher biopolymer concentrations as in the extrusion process [48]. In the extruder die, the dispersed phase is deformed, oriented along the flow and then solidified by cooling, which results in the typical anisotropic structures of extruded products.

Recently, studies on highly concentrated biopolymers structured in shear cell devices have shown that trapped air bubbles in the matrix are deformed along the flow direction and contribute to the anisotropy of the products [49,50]. However, it remains unclear whether air inclusions actually contribute to anisotropic structures in extruded plant proteins.

Despite the large number of scientific publications on the extrusion of proteins, structure formation at the mechanistic level is not yet fully understood. This paper aims to investigate both the influence of changes in protein–protein interactions, i.e., the theory of protein alignment, and of a multiphase system on the anisotropic structures formed in HME of plant proteins.

The model protein used in this study is soy protein isolate (SPI). Soy protein is by far the most studied plant protein in extrusion [51] and can be classified into four groups: 2S, 7S, 11S and 15S [52]. The specific composition of these groups is influenced by different factors such as the soy bean variety, growth conditions or the preprocessing [53]. Protein-rich SPI (protein content > 90%) is commercially produced by extraction of the protein component from soybeans. Increased temperatures in the extraction process are necessary to denature nutritionally unfavorable ingredients or to increase the yield [53]. However, this can result in denaturation of the proteins and thus significant changes in reactivity and functional properties [54,55,56]. Nevertheless, many commercially available soy proteins are, to a certain extent, intentionally processed to give the material desired functionalities [57].

Various publications have dealt with HME of SPI. Different authors used SPI as a single ingredient [23,39,40,58,59,60]; others mixed SPI with wheat starch [37,61], wheat gluten [27,38,62], mechanically deboned meat [63] or soybean residue [64]. These studies are mainly based on the hypothesis that the formation of anisotropic structures is related to the changes in protein–protein interactions. The role of additional components or phases on the structure formation is not investigated or discussed.

The aim of this study is therefore to analyze the effect of extrusion processing on protein–protein interactions and the microstructure, i.e., the multiphase morphology, in soy protein extrudates under typical HME conditions. For this, the solubility in different solvent systems and the rheological properties of the proteins before and after extrusion are examined. Furthermore, the microstructure and water distribution of the extrudates are analyzed, as phases in phase-separated highly concentrated biopolymers have been shown to differ in their local water concentrations [65,66].

## 2. Materials and Methods 

### 2.1. Materials

Commercial soy protein isolate from Solae LLC (St. Louis, MO, USA) was used for this work. According to the manufacturer, it has a protein content of at least 90% on a dry matter basis. The moisture content of the raw material, 3.4% (*w/w*), was determined gravimetrically. Tap water from Karlsruhe (Germany) was added to the extrusion process. The chemicals for the protein solubility analysis were obtained from Carl Roth GmbH + Co. KG (Karlsruhe, Germany).

### 2.2. Extrusion Trials

**Figure 1:**
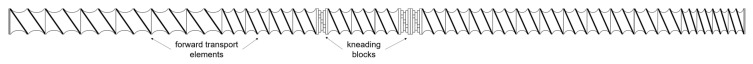
Illustration of the screw configuration in the extrusion trials.

The extrusion trials were carried out on the co-rotating ZSK 26 Mc twin-screw extruder from Coperion GmbH (Stuttgart, Germany) with a length to diameter ratio of 29 and a cooling die attached to the end of the extruder. Only forward transport and kneading elements were used in the screw configuration, as depicted in Figure 1.

The extruder barrel consists of seven sections, which can be cooled and heated individually, except for the first section, which can only be cooled. Solids were fed into the first section of the extruder barrel by a gravimetrically controlled feeder from Brabender Technology GmbH (Duisburg, Germany). Water was added at the second section of the extruder barrel by a piston membrane pump from Alldos Eichler GmbH (Pfinztal, Germany). The cooling die (380 × 30 × 6 mm) is a proprietary design of our research group and has been successfully used in earlier publications [67,68] for HME of plant proteins. The die was cooled with the temperature control liquid Thermal HL60 at 50 °C, which was supplied by a water-cooled process circulator Presto Plus LH 47, both from Julabo GmbH (Seelbach, Germany).

The screw speed was kept constant at 250 rpm. To achieve a water content of 50% (*w/w*), 5 kg/h of solids and 5 kg/h of water were added to the extruder. The temperatures of the barrels were specifically adjusted so that predefined material temperatures (124 and 135 °C) were reached right before entering the cooling die. The temperature profiles for both cases are shown in Table 1.

### 2.3. Cryo-Imaging

The microstructure was analyzed by cutting the frozen samples transversely to the flow direction (front view) with a cryo-microtome from Leica Biosystems GmbH (Nussloch, Germany). The cooling room temperature of this instrument was −14 °C, while the temperature of the sample holder was −12 °C. For cutting, the extrudates were cut in half along the flow direction and embedded in FSC 22 Blue Frozen Section Media, a sectioning medium from Leica Biosystems GmbH (Nussloch, Germany), which is liquid at room temperature but becomes solid at low temperature. This was intended to ensure product stability on the sample holder while sectioning and was tested to have no influence on the product microstructure. Then, 40-µm sections were cut from the extrudate until smooth cut surfaces were achieved. Immediately after cutting the extrudate in the cryo-microtome, the sections were discarded and photos of the cut surface were taken.

### 2.4. Micro-CT

To prepare the samples for X-ray measurements in the micro computed tomograph (micro-CT), the extrudates were freeze-dried with a freeze dryer on a small production scale with a horizontally arranged steel tube of 2.5-m length and 0.9-m diameter from the company Becker Technologies GmbH (Eschborn, Germany). Freeze drying was performed with an absolute pressure of 0.3 mbar and a plate temperature of 30 °C.

The dried samples were then analyzed with a custom-made cone beam geometry scanner of the Institute of Photon Science and Synchrotron Radiation (IPS) of the Karlsruhe Institute of Technology (KIT). In this device, samples of different sizes can be measured. Depending on the distances between object, radiation source and detector, a certain magnification can be achieved with cone geometry. Each measurement consists of a 360° rotation of the samples in the X-ray beam between the radiation source and detector with a projection of 0.176° each, resulting in 2048 projections over the entire measurement. The scanner is described in more detail elsewhere [69,70]. The image processing (noise filtering and segmentation) was performed with ImageJ2 over the distribution Fiji.

### 2.5. Protein Solubility

For the determination of changes in protein–protein interactions, raw material and extruded samples were dissolved in deionized water and two different buffer systems which are known to selectively disrupt interactions [68]. Non-covalent interactions were disrupted with a non-reducing buffer prepared with 0.132 M sodium dihydrogen phosphate, 0.068 M disodium hydrogen phosphate, 0.05 M sodium chloride, 0.0173 M sodium dodecyl sulfate (SDS) and 8 M urea. The disulfide bonds were broken with a reducing buffer prepared by adding 0.01 M dithiothreitol (DTT) to the non-reducing buffer. The pH values of the non-reducing and reducing buffer were adjusted to 7.0 with 0.1 M sodium hydroxide (NaOH).

The samples, both extrudates and raw material, were pre-dried for at least 72 h at 25 °C in room air, ground, classified to a particle size < 280 µm and vacuum-dried at 25 °C to a constant mass. Then, 10 mg of the sample was mixed with 19 g buffer, vortexed for 30 s and dissolved for at least 24 h at room temperature on a rotary shaker from Edmund Bühler GmbH (Bodelshausen, Germany) at 200 rpm. The samples were then centrifuged in a 5920 R centrifuge from Eppendorf AG (Hamburg, Germany) at 4301× *g* for 50 min. The supernatant was used for further analysis. 

To determine the protein concentration in the supernatant, the absorbance at 280 nm was analyzed in an Evolution 201 spectrophotometer from Thermo Fisher Scientific Inc. (Waltham, MA, USA). The relative protein concentration was calculated using a calibration curve with bovine serum albumin (BSA) as standard. Solubility and absorbance measurements were repeated four and three times, respectively, resulting in a total of twelve absorbance values per sample.

A gravimetrical determination of the solubility of raw material in deionized water was performed by pre-drying the material for at least 72 h at 25 °C in room air. Then, 0.5 g of the sample was mixed with 40 g of deionized water, vortexed for 30 s and dissolved for at least 24 h at room temperature on the rotary shaker at 200 rpm. The samples were then centrifuged in the 5920 R centrifuge at 4301× *g* for 50 min. The supernatant and centrifugate were separated and weighed individually. The supernatant was dried at 105 °C for at least 72 h to remove the water. The remaining solid was weighed, and the solubility was calculated from the weight quotient of solid in the supernatant and the overall weighed-in solid.

### 2.6. Rheological Measurements

**Figure 2:**
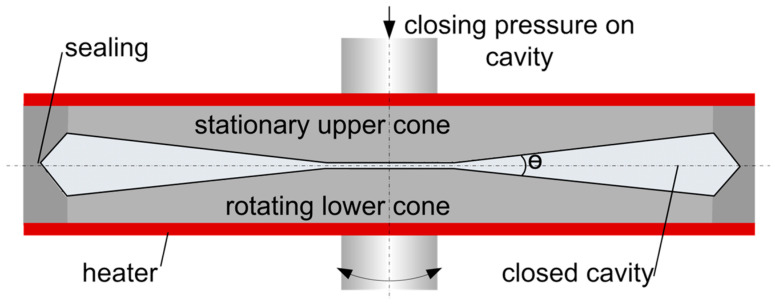
Closed cavity rheometer used for rheological measurements.

To determine the rheological properties, samples were analyzed in a closed cavity rheometer RPA flex from TA Instruments, Inc. (New Castle, DE, USA); see Figure 2. This instrument is described in detail elsewhere [71,72] and will only be briefly described here. The material is first placed between the two cones. The downward movement of the upper cone and the application of a closing pressure creates a closed cavity for the material, which prevents water evaporation and allows measurements at high temperatures. The lower cone exerts a sinusoidal rotary deformation and the resulting force is monitored, from which the resulting rheological properties can be calculated.

For the investigation of the influence of extrusion processing on the rheological properties, the samples, both extrudates and raw material, were prepared by pre-drying for at least 72 h at 25 °C in room air, grinding, classifying them to a particle size < 450 µm and drying them to constant mass. The dried powder was then rehydrated with a Thermomix from Vorwerk (Wuppertal, Germany) to the water content (50% (*w/w*)) used in the extrusion process. 

For the determination of the temperature dependence of rheological properties, raw material was mixed with water in the Thermomix to achieve a water content of 50% (*w/w*).

The doughs were stored under vacuum at 4 °C for at least 12 h prior to the measurements to ensure uniform water distribution and hydration. 

To investigate the influence of extrusion processing on the material, the complex viscosity η* as a function of angular frequency was determined by frequency sweeps in the linear viscoelastic range at a constant amplitude of 0.98%. The measurements were repeated six times.

The temperature dependence was determined by measuring the stationary complex viscosity of the raw material dough at a constant angular frequency of 6.28 rad/s and an amplitude of 0.98% at temperatures from 50 to 120 °C. Above 120 °C, the material viscosity was too low to be measured by the torque sensor; therefore, the rheological measurements were not feasible. These measurements were also repeated six times.

### 2.7. Statistical Analysis

The software OriginPro (version 9.6.) from OriginLab Corporation (Northampton, MA, USA) was used for statistical analysis. The changes in protein–protein interactions and rheological properties through extrusion processing were evaluated by one-way ANOVA using Scheffé’s test for comparison of means with a probability of *p* < 0.01 to identify significant differences. 

## 3. Results

### 3.1. Product Structure of Extruded Soy Protein

In the extrusion trials with SPI, screw speed and mass flow were held constant. The water content was set to 50% (*w/w*), which is typical for HME applications [73]. The sections of the barrels were heated decisively to induce material temperatures of 124 and 135 °C before entering the cooling die. Other publications on HME of SPI reported the formation of anisotropic structures for similar conditions—Chen et al. [59] extruded SPI at 28–60% and Fang et al. [40] at 50% water content.

**Figure 3:**
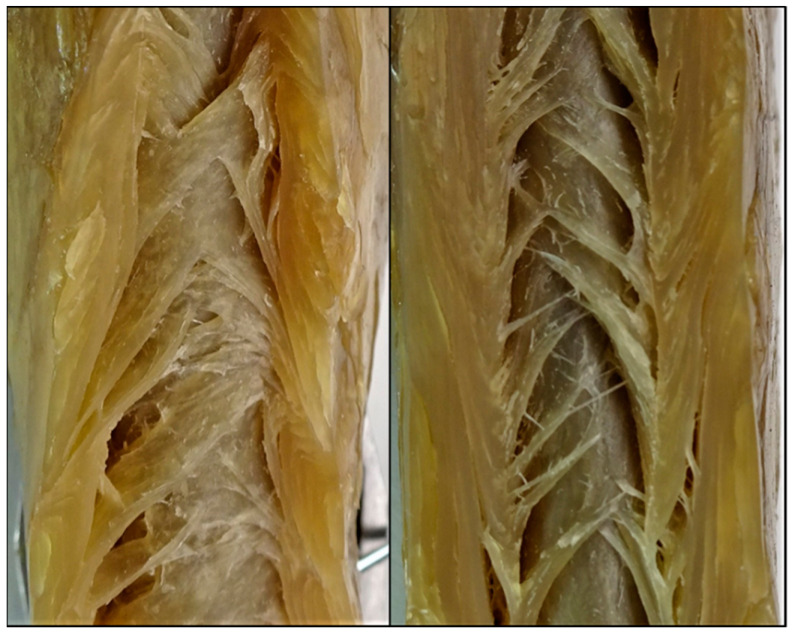
Product structure of extruded soy protein isolate (SPI) with material temperatures of 124 °C (**left**) and 135 °C (**right**) before cooling die. Water content (50%), screw speed (250 rpm) and mass flow (10 kg/h) were held constant. Increase in material temperature was induced by increasing the barrel temperature.

The product structures resulting from extrusion trials are depicted in Figure 3.

At both temperatures, a distinct anisotropic product structure is visible. Although many publications on HME exist, only few of them include pictures of the product structure, which makes comparison difficult. Nevertheless, similar product structures have been reported in HME of pea protein isolates [28], blends of SPI, wheat gluten and corn starch [41] and blends of soy protein concentrate (SPC) and microalgae [74]. 

An early study on HME of plant proteins reported that it was not possible to create anisotropic structures with SPI and a second component would be necessary (e.g., wheat gluten) for this purpose [75]. However, as shown in the present case and other publications [23,40,59], SPI can be sufficiently structured through HME without any additional component.

With increasing the temperature from 124 to 135 °C, higher anisotropy, i.e., thinner structures, was achieved. The dependency of the product structure and/or texture on the process temperature has been reported in other publications on HME of plant proteins as well [23,25,28,59,67]. However, the role of temperature remains unclear, as some other studies have shown that temperature does not have a significant effect on product structure [60].

### 3.2. Changes in Protein–Protein Interactions through Extrusion Processing

**Figure 4:**
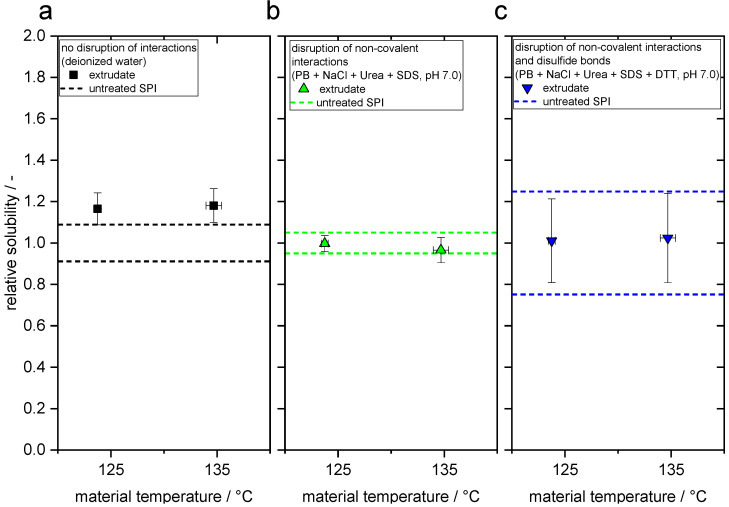
Relative solubility (sample solubility/raw material solubility) of the extrudates in different solvent systems as a function of temperature. From left to right: deionized water, non-reducing solvent and reducing solvent. PB = phosphate buffer, SDS = sodium dodecyl sulfate, DTT = dithiothreitol.

The changes in protein–protein interactions can be detected by determination of the protein solubility in different solvent systems. For this purpose, the solubility of raw and extruded protein was investigated in deionized water and two buffers which are known to selectively break up protein–protein interactions. The results of the solubility measurements are depicted in Figure 4.

The relative solubility is the quotient of sample solubility and raw material solubility. The solubility in deionized water increases significantly (*p* < 0.01) through extrusion processing at both temperatures. These minor changes suggest that some non-covalent interactions (i.e., electrostatic and hydrophobic interactions, hydrogen bonds) are disrupted in the process. However, there is no significant (*p* > 0.01) difference between both temperatures. The gravimetrically determined solubility of the raw material in deionized water is 6.2% (*w/w*). In general, soy protein isolates are reported to have a relatively low water solubility [56], which can probably be traced back to the thermal stresses occurring in isolation of proteins from the soy bean [52]. After disruption of non-covalent interactions in non-reducing buffer, the solubilities of raw material and both extruded samples are not significantly different (*p* > 0.01). In reducing buffer, which disrupts disulfide bonds, the solubilities of the raw material and both extruded samples are also not significantly different (*p* > 0.01). This suggests that covalent interactions such as disulfide bonds are not influenced by the extrusion process, although this effect has been proposed to play a very important role as a prerequisite for anisotropic structure formation [76]. 

Similar findings on the effect of extrusion processing were reported by a study from Chen et al. [39], in which SPI was extruded at 60% water content and a maximum barrel temperature of 150 °C. Samples of extruded SPI were collected along the screw after a dead-stop. In both non-reducing and reducing buffer (pH = 7.6), only minor changes of protein solubility occurred along the screw. However, the authors found distinct changes in solubility in all buffer systems when extruding at a lower moisture content (28%). 

Pietsch et al. [68] published a study on HME of SPC with similar process conditions and the same extruder setup as in the present work and also reported that only minor changes in protein–protein interactions are induced through extrusion processing.

Contrary to the previous two studies, in the HME of SPI blended with wheat starch [37] and with wheat gluten and wheat starch [38], the effect on protein–protein interactions was much more pronounced; for example, protein solubility in phosphate buffer decreased by about 70% and 90%, respectively. In another study, HME of SPI led to an increase of about 100% in the molecular weight [60].

As in the present case, the extrusion/material temperature is reported to have no or only a minor effect on the protein–protein interactions of SPI and SPC in HME [37,59,68].

In general, many authors have dealt with the influence of extrusion processing on the protein–protein interactions of soy proteins. In LME (i.e., up to 40% water content), changes in interactions were reported in almost all studies with all types of soy protein sources (defatted soy flour, SPC and SPI) and different experimental setups [29,30,31,32,33,34,35,36]. However, as mentioned before, findings are not consistent in HME. These sometimes contrary findings can be explained by two phenomena.

First, the viscosities of the materials are higher at lower moisture content. On the one hand, this leads to higher temperatures due to viscous dissipation. On the other hand, the shear stresses, which are a function of the local shear rate and viscosity, are much higher. Significant influence of shear stresses on molecular structure of extruded SPI was, for instance, shown by Fang et al. [40]. Thermomechanical stresses in LME are therefore generally higher than in HME, which could explain the changes in protein–protein interactions often reported during LME of plant proteins.

Secondly, the raw materials used in studies on HME of soy proteins were obtained from different sources. It has already been shown that SPIs from different manufacturers have different functional properties [57,77] and extrusion behaviors in both LME and HME [26,27]. Furthermore, SPI undergoes more intense processing steps during extraction and isolation than SPC or defatted soy flour [53]. 

It is therefore assumed that the SPI in the present case has experienced higher thermal and mechanical stresses during pre-processing in comparison with the SPI from the other studies, which results in a higher denaturation degree and, therefore, lower reactivity. These higher stresses could have been applied whether coincidentally or intentionally to modify functional properties such as water absorption or gelling properties [78]. Additionally, it is assumed that the covalent protein–protein interactions formed during pre-processing such as disulfide and isopeptide bonds, are resistant against the thermomechanical stresses occurring in the present extrusion process.

Although the solubility results in the present case suggest that minor changes of non-covalent interactions and no changes of covalent interactions took place in the process, they must be interpreted carefully. For instance, continuous changes (e.g., simultaneous disruption and creation of bonds) in the interactions during the process may not be reflected in the solubility when comparing raw material and final extrudate. Furthermore, interactions could be influenced without actually having an influence on the solubility in any of the solvent systems. This seems to be quite of importance, since even in reducing buffer, more than 50% (*w/w*) remain insoluble and it is very unlikely that the protein–protein interactions in this insoluble fraction are not influenced by the solvent.

More comprehensive information on protein–protein interactions and the molecular weight distribution of all protein fractions can be obtained by analyzing the rheological properties, which are a function of the molecular structure of the entire protein [79]. For example, if insoluble protein aggregates break up but remain insoluble, this is not reflected in the previously discussed solubility of the proteins but would lead to a decrease in viscosity as the molecular weight decreases.

### 3.3. Changes in Rheological Properties through Extrusion Processing

**Figure 5:**
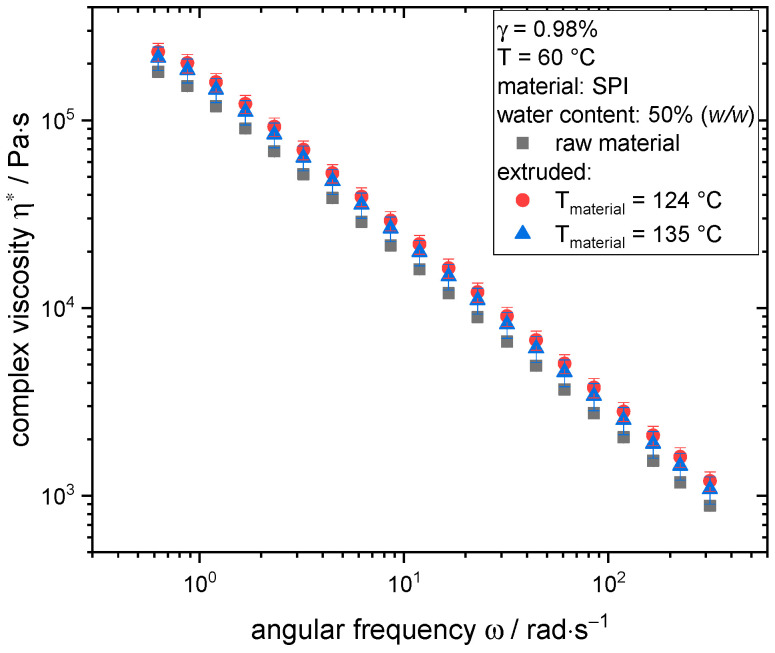
Frequency sweeps (γ = 0.98%) of raw material and extruded samples at T = 60 °C and water content of 50% (*w/w*).

To determine the rheological properties, the raw material and extruded samples were analyzed with frequency sweeps at 60 °C. The results are depicted in Figure 5; complex viscosity η* is plotted as a function of the angular frequency ω.

For all investigated samples, a decrease in the complex viscosity with increasing angular frequency is observed. This behavior is typical for highly concentrated plant proteins and was reported before for SPI [72,80]. 

Comparing the curves of extruded samples and raw material, there is a slight difference between the raw material and the sample extruded at 124 °C. However, no significant (*p* > 0.01) differences exists between raw material and sample extruded at 135 °C as well as between both extruded samples. From the experience on the method, the minor change at 124 °C is assumed to be negligible. This becomes more obvious when compared to other studies; in systems with reactive proteins such as wheat gluten [71] or whey protein [81], typical extrusion treatment can lead to up to a ten-fold increase in viscosity.

Rheological properties of soy flour dough [32] and highly concentrated SPI [82,83] have been shown to be influenced by heat and shear treatment. However, it seems that in the present case, extrusion processing has only a negligible influence on the rheological properties. 

### 3.4. Influence of Temperature on Rheological Properties of Soy Protein 

**Figure 6:**
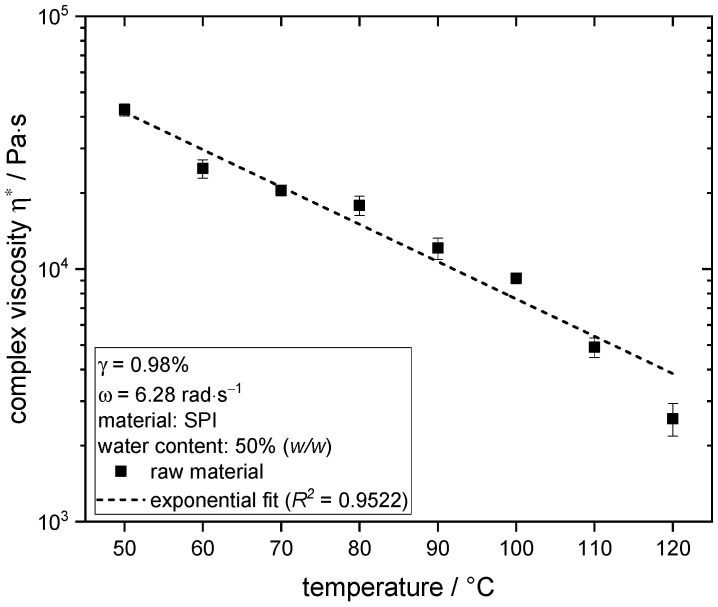
Complex viscosity of raw material SPI at 50% (*w/w*) water content as a function of temperature, determined at constant γ = 0.98% and ω = 6.28 rad·s^−1^.

The complex viscosity of the raw material was determined as a function of the temperature, and the results are depicted in Figure 6.

The complex viscosity of the material decreases exponentially with increasing temperature, which has also been shown before for defatted soy flour [84] and is typical for most liquid and molten polymers [85]. This can be explained by the increased mobility of soy proteins with increasing temperature. Although the measurements were performed at maximum 120 °C, it can certainly be expected that the effect of temperature on viscosity can be extrapolated to the slightly higher temperatures of the extrusion trials. The exponential fit allows the estimation of a viscosity decrease of about 31% with the temperature increase from 124 to 135 °C.

These results therefore suggest that the changes in product structure induced by the temperature increase from 124 to 135 °C in the extrusion process might be due to the increased mobility of molecules and the resulting changes of rheological properties. 

### 3.5. Influence of Processing Conditions on the Extrudate Microstructure 

**Figure 7:**
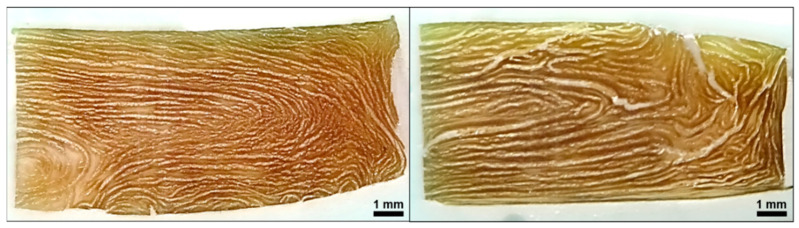
Microstructures of frozen extrudates of SPI extruded at 50% (*w/w*) moisture content and material temperatures of 124 °C (**left**) and 135 °C (**right**) before entering the cooling die. The front view is displayed. Extrudates are embedded in sectioning media.

The frozen extrudates were cut in half along the flow direction, embedded in sectioning medium and cut transversely to the flow direction (frontal view) to analyze the microstructure; pictures of the cut extrudates can be seen in Figure 7.

The left edges of both samples correspond to the point where the extrudates were cut in half (symmetry axis), while the top, bottom and right edges were in contact with the walls of the cooling die. For both extrudates, a distinct multiphase system is present. A brownish continuous matrix covers most of the cut surface. This continuous phase surrounds a very light, almost white-colored dispersed phase, with a crystalline appearance. At the lower temperature (left), this light-colored phase is finely dispersed, whereas increasing the temperature seems to result in more coarse structures. This change in phase morphology is expected to be responsible for the different product structures, as depicted in Figure 3.

A dispersed phase is apparent, although no second protein or polysaccharide component has been added. However, Tolstoguzov [44] reported that phase separation could also take place within the same protein having different fractions and/or conformations. 

It has also been described by Tolstoguzov that the water redistributes through phase separation, which leads to a water-rich and a water-poor phase. This phenomenon has already been reported in highly concentrated proteins structured via simple shear flow [65,66] and suggested for HME of pea protein isolate [86]. 

Our results strongly suggest that water redistribution has occurred here as well, which means that the light dispersed phase has a relatively high water content and its white appearance originates from ice crystals. 

### 3.6. Water Distribution in Soy Protein Extrudates

To confirm the hypothesis that a water-rich and a water-poor (i.e., protein-rich) phase were created, the sample extruded at 124 °C was investigated before and after freeze-drying via X-ray scanning. Different to other drying methods, freeze-drying has been shown to preserve structural features of biological samples very well [87] and to reveal anisotropic structures of extrudates from HME [86,88]. Through X-ray measurement, local density differences can be visualized, as the X-rays are absorbed differently. 

The X-ray measurement of the extrudate before freeze-drying at room temperature was not able to detect any density differences in the product; a homogeneously white picture of the extrudate was obtained. It is assumed that the differences in water concentration between the phases, i.e., the difference of contrast between the phases, is not high enough to be visualized. 

If air bubbles were incorporated through extrusion processing, they should be detectable and visible in the X-ray measurements due to the very low density of air compared to the protein system. As they were not visible, it is assumed that entrapped air bubbles do not contribute to the anisotropic structures in the extrudates, contrary to systems processed in shear cells [49,50].

**Figure 8:**
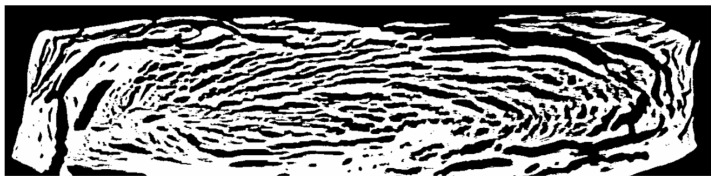
Microstructure of freeze-dried SPI extrudate, obtained with X-ray measurements. Sample was extruded at 50% (*w/w*) moisture content and material temperature of 124 °C before entering the cooling die. Front view is displayed. Flow direction is directed out of the image plane towards the reader.

The front view of freeze-dried extrudates obtained through X-ray measurement is depicted in Figure 8.

Through freeze-drying, the overall structure is fixed and water sublimates, which minimizes the influence of the drying step on the microstructure. Water-rich regions turn into regions which predominantly consist of air, whereas water-poor, i.e., protein-rich, regions result in predominantly solid regions. In the displayed front view of the extrudate, black pixels correspond to air and white pixels correspond to solid areas (i.e., high density).

In comparison with Figure 7, which is the same frontal view of the extrudate, a very similar overall appearance of phase morphology can be seen. It is therefore concluded that the light, crystalline phase in Figure 7 actually is water-rich and the brown phase is water-poor. 

**Figure 9:**
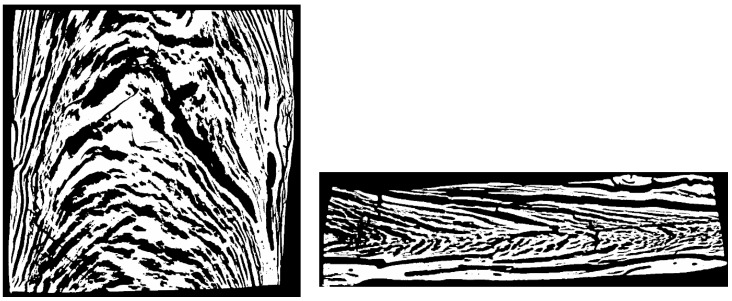
Microstructure of freeze-dried SPI extrudate, obtained with X-ray measurements. Sample was extruded at 50% (*w/w*) moisture content and material temperature of 124 °C before entering the cooling die. Top view (**left**) and side view (**right**) are displayed. Flow direction is directed from bottom to top for the top view and left to right for the side view.

It can also be seen that the dispersed phase shows some kind of orientation. This orientation of the dispersed phase is clearly visible in the top and side views of the extrudate as well (Figure 9). 

The top and side views show that the dispersed phase is oriented along the flow in the die. The typical flow profiles of a Hagen–Poiseuille flow are visible. Similar flow profiles have been obtained in numerical simulations of a model HME process [86] and reported in extrudates [89,90]. Flow characteristics in the cooling die, therefore, must play an important role in the morphology of the multiphase system.

## 4. Discussion

Two main mechanisms, which have been under discussion up to today, are used to explain the formation of anisotropic structures in HME of plant proteins: the alignment of protein molecules or the existence of a multiphase system. 

Many studies on HME have focused on the investigation of molecular structure, e.g., determination of changes in protein–protein interactions or in the molecular weight. This is most likely due to the assumption that possibly aligned protein molecules must be stabilized in the final product by creation of new interactions [76], often described as “cross-linking”. Especially disulfide bonds and hydrophobic interactions are reported to be the most important for this stabilization. In the present case, the results of the solubility and rheological measurements suggest that only a negligibly small amount of these new interactions is created in the process. It is therefore assumed that the mechanism of aligning protein molecules and a subsequent stabilization is not required to achieve an anisotropic structure. This assumption is supported by results from the literature, for example, in a study with LME of SPI [91]. Measurements with X-ray diffraction and infrared-spectroscopy revealed that no changes in protein–protein and protein–polysaccharide interactions occurred and that no dominant orientation of the proteins existed. The anisotropy could be attributed only to the orientation of the dispersed phase. Furthermore, Chen et al. [39] reported anisotropic structures in HME of SPI, although only minor changes in protein–protein interactions took place. 

Instead, the microstructural analysis in this study revealed a distinct multiphase system in the extrudates. Through the extrusion process, a water-rich dispersed phase was created, surrounded by a water-poor, i.e., protein-rich, continuous phase. The mechanism of water redistribution in highly concentrated, phase-separated biopolymer melts has been proposed by Tolstoguzov [44] from observations in biopolymer solutions and reported in highly concentrated biopolymer structures in shear cells [65,66]. However, this phenomenon was shown for multicomponent systems, i.e., mixtures of raw materials (SPI and wheat gluten and pea protein isolate and wheat gluten). In the present case, only one raw material (SPI) was used.

It is therefore suggested that the phase separation took place between different fractions of the SPI, e.g., the water-soluble and water-insoluble protein fractions. The mechanism of phase separation between biopolymers with different hydrophilicities/hydrophobicities has already been described [47]. Only a low percentage of the SPI is water-soluble (6.2% (*w/w*)); the predominant part is water-insoluble. Furthermore, more than 50% of the protein remains insoluble in reducing buffer. Additionally, the comparison with rheological properties of other plant proteins shows that viscosity under the same conditions is relatively high. This leads us to the assumption that highly aggregated proteins with a high molecular weight and low solubility, presumably stabilized via isopeptide bonds, were created in the pre-processing of SPI. These insoluble proteins could form the continuous phase, while the soluble proteins could be located in the water-rich dispersed phase.

In such multiphase systems, the rheological properties and flow characteristics play an important role on the morphology development. The flow characteristics are a function of the mass flow, geometrical parameters, temperature distribution and rheological properties. In the present case, temperature increase led to decreased viscosity, which explains why the morphology is affected by temperature increase. However, not only viscosity plays a role in multiphase systems; other rheological properties such as elasticity must have a significant influence as well and should be considered in the future [72]. 

Although the aligned proteins seem not to be the origin of anisotropic structures, the molecular structure remains an important factor in the multiphase systems formed in extruded plant proteins. It influences the hydrophilicity/hydrophobicity and, therefore, the water distribution in the system and determines the rheological properties and immiscibility of proteins. In the case of reactive proteins, investigations of process-related changes in protein–protein interactions should, therefore, be connected with a determination of rheological properties and water solubility/absorption.

This study suggests that multiphase dynamics must be considered for successful process and product design in the HME of plant proteins. The investigation of molecular structure is important as it influences the rheological properties and water dynamics in the systems; however, limitations of the method, especially regarding the insoluble proteins, in predicting extrusion behavior should be kept in mind. The determination of water distribution seems to be an effective tool to investigate and evaluate anisotropic structures in extruded plant proteins. Functional properties such as water solubility or water absorption capacity, which influence water redistribution, should be considered when evaluating raw materials and designing extruded meat analogues from plant proteins.

## 5. Conclusions

The HME of a plant model protein, SPI, led to anisotropic product structures that are also dependent on temperature. Only negligible small changes of protein–protein interactions were observed. Cryo-imaging of frozen samples and X-ray analysis of freeze-dried extrudates revealed a pronounced multiphase system. A water-rich dispersed phase was surrounded by a continuous water-poor, i.e., protein-rich, phase. Changes in the product structure through temperature increase contributed to changed rheological properties due to an increased mobility of the molecules. The importance of the influence of flow characteristics on the product structure was clearly evident from the microstructural analysis, as the multiphase morphology was shaped like a typical Hagen–Poiseuille flow profile. 

The results show that the formation of anisotropic structures in the extruded plant protein results from a multiphase system with a locally differing water concentration. The analysis of this system requires advanced and novel methods for a mechanistic investigation of the structure formation. Cryo-imaging and X-ray measurement of freeze-dried extrudates seem to be convenient and promising methods for this purpose.

## Figures and Tables

**Table 1:** Temperature profile of extruder barrel sections for the cases of a material temperature of 124 °C and 135 °C.

Setting No.	1	2
Material temperature/°C	124	135
Temperature barrel 2/°C	40	40
Temperature barrel 3/°C	60	60
Temperature barrel 4/°C	80	90
Temperature barrel 5/°C	110	130
Temperature barrel 6/°C	123	135
Temperature barrel 7/°C	124	140

The samples were taken after the extruder responses had been stationary for at least three minutes. To analyze the product structure, the samples were photographed immediately after removal from the process. To analyze the microstructure, the extrudates were frozen immediately after the extrusion process and stored under vacuum at −18 °C until further examination.
